# Diabetes and the social, biologic, and behavioral determinants of endometrial cancer in the United States

**DOI:** 10.1186/s12885-024-12192-y

**Published:** 2024-04-29

**Authors:** Nour Massouh, Ayad A. Jaffa, Miran A. Jaffa

**Affiliations:** 1 Epidemiology and Population Health Department, Faculty of Health Sciences, American University of Beirut, Beirut, Lebanon; 2 Department of Biochemistry and Molecular Genetics, Faculty of Medicine, American University of Beirut, Beirut, Lebanon; 3 Division of Endocrinology, Diabetes & Metabolic Diseases, Department of Medicine, Medical University of South Carolina, Charleston, SC USA

**Keywords:** Behavioral risk factor Surveillance System (BRFSS), Diabetes, Endometrial cancer, Obesity, Race, Social determinants of Health, Weighted Chi-square test, Weighted logistic regression

## Abstract

**Background:**

Endometrial cancer is one of the most common types of cancer that affects women’s reproductive system. The risk of endometrial cancer is associated with biologic, behavioral and social determinants of health (SDOH). The focus of the work is to investigate the cumulative effect of this cluster of covariates on the odds of endometrial cancer that heretofore have only been considered individually.

**Methods:**

We conducted a quantitative study using the Behavioral Risk Factor Surveillance System (BRFSS) national data collected in 2020. Data analysis using weighted Chi-square test and weighted logistic regression were carried out on 84,118 female study participants from the United States.

**Results:**

Women with diabetes mellitus were approximately twice as likely to have endometrial cancer compared to women without diabetes (OR 1.54; 95%CI: 1.01–2.34). Biologic factors that included obesity (OR 3.10; 95% CI: 1.96–4.90) and older age (with ORs ranging from 2.75 to 7.21) had a significant increase in the odds of endometrial cancer compared to women of normal weight and younger age group of 18 to 44. Among the SDOH, attending college (OR 1.83; 95% CI: 1.12-3.00) was associated with increased odds of endometrial cancer, while renting a home (OR 0.50; 95% CI: 0.28–0.88), having other arrangements (OR 0.05; 95% CI: 0.02–0.16), being divorced (OR 0.55; 95% CI: 0.30–0.99), and having higher incomes ranging from $35,000 to $50,000 (OR 0.35; 95% CI: 0.16–0.78), and above $50,000 (OR 0.29; 95% CI: 0.14–0.62), were all associated with decreased odds of endometrial cancer. As for race, Black women (OR 0.24; 95% CI: 0.07–0.84) and women of other races (OR 0.37; 95% CI: 0.15–0.88) were shown to have lower odds of endometrial cancer compared to White women.

**Conclusion:**

Our results revealed the importance of adopting a comprehensive approach to the study of the associated factors of endometrial cancer by including social, biologic, and behavioral determinants of health. The observed social inequity in endometrial cancer among women needs to be addressed through effective policies and changes in social structures to advocate for a standardized healthcare system that ensures equitable access to preventive measures and quality of care.

**Supplementary Information:**

The online version contains supplementary material available at 10.1186/s12885-024-12192-y.

## Background

 Endometrial cancer is a type of cancer that develops in the tissue lining of a woman’s uterus [1], with associated symptoms of pelvic pain, and excessive, prolonged, or irregular bleeding between menstrual periods [2]. Endometrial cancer is currently the 7th most common cancer and the 14th leading cause of death among women in the world [3] and in the United States (US) with 63,000 newly reported cases in the year 2022 [4, 5].

Diabetes, a chronic disease characterized by high levels of glucose in the bloodstream [6], has been reported to be associated with endometrial cancer [7–15]. In this respect, a systematic review that assessed the relationship between diabetes mellitus (DM) and endometrial cancer, analyzed 29 eligible cohort studies, and reported a summary relative risk of 1.89 for women with DM compared to women without DM [15]. In addition, this study has also reported a summary incidence rate ratio of 1.61 for women with versus without DM [15]. Another research article reported results from a systematic review and meta-analysis conducted on 22 cohort and case-control studies identified 14 studies that detected significant association between DM and endometrial cancer. This article showed that diabetes was significantly associated with endometrial cancer with relative risk of 1.72, a summary relative risk of 1.56 in 9 of the cohort studies, and 1.85 in 13 case control studies [13]. With respect to diabetes-attributed mortality rate among women with endometrial cancer, reported statistics highlighted that the risk of disease-specific mortality was 32% times higher among women with versus without diabetes [10, 15].

The relationship between diabetes and endometrial cancer may be attributed to shared biologic factors such as obesity and age [16, 17]. In this respect, obesity has been identified as a major risk factor for both diseases [16, 17], due to its association with insulin resistance that results in type 2 diabetes [17]. Obesity is also linked to the excess body fat that leads to hormonal imbalances and elevation in the levels of estrogen unopposed by progesterone which can then lead to early stages of endometrial cancer formation [13, 14, 18]. With respect to age, it was established that older age is a risk factor for both diabetes and endometrial cancer [5, 16]. In this regard, recent statistics indicated that the incidence and mortality rates of endometrial cancer increase with age [5], particularly among women aged 55 or more [19], with approximately 80% of new cases and 91.3% of endometrial cancer-specific deaths in the US occurring within this age group [19]. The effect of age on endometrial cancer may be ascribed to the hormone replacement therapy. In specific, estrogen-only replacement therapy [20], that is recommended for women going through the menopausal transition typically occurring between the ages of 45 and 55 [21], was shown to double the risk of endometrial cancer [22].

Behavioral factors, such as cigarette smoking and alcohol consumption, were also identified as factors that may affect endometrial cancer. In this regard, some epidemiological studies have determined an association between smoking and a decreased risk of endometrial cancer [23–25], possibly due to its anti-estrogenic effect through altering hormone metabolism [23] and consequent weight loss [23, 24]. Emerging evidence has also suggested that there may be a potential relationship between alcohol consumption and endometrial cancer [26–28]. This relationship may be attributed to the fact that alcohol can raise the levels of estrogen in the blood which then increases the risk of endometrial cancer [27].

In conjunction with the aforementioned biologic and behavioral factors, racial and social disparities in the distribution of endometrial cancer have been marked across the different socioeconomic classes. More specifically, various indices of socioeconomic status, referred to as social determinants of health (SDOH), including marital status, level of education, and healthcare coverage, were shown to have an effect on endometrial cancer [29–32]. For instance, unavailability of healthcare coverage and lower levels of education were identified as risk factors for endometrial cancer in several studies [29–31]. Specifically, women with no healthcare coverage were shown to have higher rates of advanced-stage diagnosis of endometrial cancer and unequal access to treatment compared to women with healthcare coverage [31]. Conversely, being married was associated with lower mortality rates compared to being divorced (Hazard Ratio HR 1.19), widowed (HR 1.22), or never married (HR 1.23) [32].

Disparities in endometrial cancer were also highlighted among women of different races whereby White women are more frequently diagnosed with endometrial cancer compared to women of other races [33]. However, Black women tend to have more advanced stages and aggressive tumors of endometrial cancer compared to White women [33, 34]. Similarly, the risk of death attributed to endometrial cancer among Black women was shown to be 2.5 times higher than that of White women, and the disease-specific mortality rate of endometrial cancer among women of Black race was reported as 9.2 per 100,000 compared to 4.6 per 100,000 for women of White race [35]. These statistics reflect a higher prevalence in the incidence of endometrial cancer among White women, but increased mortality rates among Black females. The aforementioned racial disparities in endometrial cancer incidence and disease-specific mortality rates may be credited to embedded differences in the exposure to risk factors, comorbidities, and unequal access to health care providers, diagnosis, and treatment services among the different racial groups [31].

Despite that the effects of all of these social, biologic, and behavioral determinants of health were independently reported on diabetes and cancer, no study has offered a comprehensive understanding of the collective effect of these determinants of health, along with diabetes on the risk of endometrial cancer among women. Therefore, the objective of this study is to address this gap in knowledge by investigating, for the first time, the association between this cluster of predictors and endometrial cancer using a nationally representative sample of American women adopted from the Behavioral Risk Factor Surveillance System (BRFSS). We hypothesize that the occurrence and development of endometrial cancer are increased by diabetes and indices of social determinants of health. The acquirement of this knowledge is important in order to develop new strategies to alleviate the burden of endometrial cancer, by addressing the contribution of biologic, social and racial determinants on the incidence, development and progression of endometrial cancer.

## Methods

### Study population and sampling

Our study was based on the 2020 BRFSS, a national surveillance system updated yearly by the Centers for Disease Control and Prevention (CDC) to collect information from residents in the US across all the states [36]. This CDC-BRFSS survey included questions on health-related risk behaviors, chronic health conditions, and the use of preventive services. However, not all states allowed administering questions on cancer survivorship and the specific type of cancer. Therefore, in our analysis, we included participants from the following 22 states that were asked about the type of cancer: Arizona, Connecticut, Delaware, Georgia, Hawaii, Indiana, Louisiana, Massachusetts, Michigan, Mississippi, Missouri, Montana, New Jersey, New Mexico, North Carolina, Rhode Island, South Dakota, Utah, Vermont, Virginia, Wisconsin, and Guam. The CDC adopted a multistage cluster design to randomly select adult participants (aged 18 or more), hence providing a nationally representative sample [37]. We excluded participants that reported having pre-diabetes or borderline diabetes, had diabetes only during pregnancy, or had missing data concerning their diabetes status. Male participants were also excluded. Therefore, our study population was comprised of a total sample of 84,118 female participants from the aforementioned 22 states in the US.

The flowchart below includes the details of how the final sample size was reached:


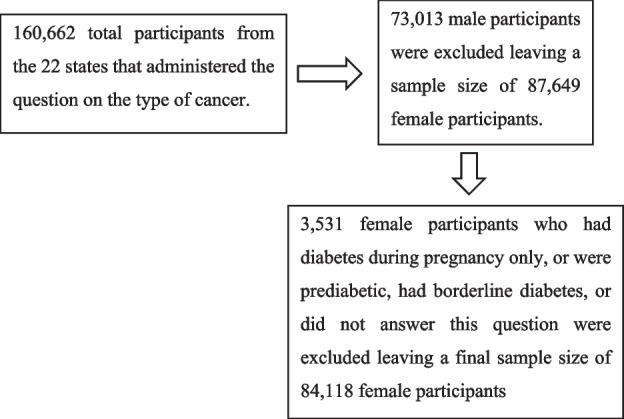


### Concepts and measures

The main dependent variable in the study is endometrial cancer dichotomized into two levels indicating the presence or absence of this cancer type among women. Endometrial cancer was determined through the question in the BRFSS which asked about the type of cancer the participant was most recently diagnosed with. If women reported that endometrial cancer was the most recent type of diagnosed cancer then our outcome was set to reflect the presence of endometrial cancer (endometrial cancer = yes); otherwise endometrial cancer was set as not present (endometrial cancer = no). Age of diagnosis with cancer was also reported. For participants with more than one type of cancer, the age of diagnosis with the first type of cancer was the one that was recorded.

Independent variables encompass diabetes, SDOH, biologic, and behavioral factors. SDOH included homeownership, marital status, healthcare coverage, employment status, urban/rural county, education level, income level, and race. Race was categorized into White, Black, Asian, Other race, and Multiracial. Other race included American Indian, Alaskan Native, Native Hawaiian/other Pacific Islander, or women who did not identify with any of the aforementioned race categories.

Biologic factors covered age and obesity, which is determined by body mass index (BMI), and behavioral factors included smoking status and heavy alcohol consumption. The different categories of each variable are detailed in Table 1. According to the CDC classification, BMI was classified into four categories: underweight (less than 18.5 kg/m^2^), normal weight (between 18.5 kg/m^2^ and 25.0 kg/m^2^), overweight (between 25.0 kg/m^2^ and 30.0 kg/m^2^), or obese (more than 30.0 kg/m^2^) [38], and heavy drinkers were defined for women as consuming more than 7 drinks per week [36].

**Table 1:** Characteristics of the study population and crude associations with endometrial cancer

Variables	N	Weighted %	Weighted Chi-square *p*-value	Weighted Unadjusted Logistic Regression *P*-value
**Endometrial Cancer**				
No	83,162	99.6		
Yes	408	0.4		
Diabetes			<0.001*	
No	73,188	88.9		Ref
Yes	10,930	11.1		<0.001*
Among Diabetics				
Endometrial Cancer (Yes)	106	0.91		
Endometrial Cancer (No)	10,776	99.09		
**Social Determinants of Health**				
Home Ownership			<0.001*	
Own	58,970	69.3		Ref
Rent	20,056	24.2		<0.001*
Other Arrangement	4,375	6.4		<0.001*
Marital Status			<0.001*	
Married or Coupled	43,625	52.9		Ref
Divorced or Separated	13,455	14.0		0.08
Widowed	12,653	9.9		0.003*
Never Married	13,603	23.2		<0.001*
Health Care Coverage			0.11	
No	5,841	9.8		Ref
Yes	77,946	90.2		0.12
Employment Status			<0.001*	
Employed or Self-Employed	37,635	50.2		Ref
Out of Work/Unable to Work	10,846	14.8		0.004*
Homemaker/Student	8,358	14.0		0.41
Retired	25,833	21.0		<0.001*
Urban/Rural County			0.12	
Urban	73,153	92.9		Ref
Rural	9,909	7.1		0.13
Education Level			0.11	
Graduated high school	21,378	26.5		Ref
Did not graduate high school	5,131	10.5		0.73
Attended college or technical school	24,160	32.6		0.07
Graduated college or technical school	33,129	30.4		0.60
Income Level			0.18	
Less than $15,000	6,042	9.5		Ref
$15,000 to less than $25,000	10,910	16.6		0.34
$25,000 to less than $35,000 $35,000 to less than $50,000	6,845 9,141	9.8 13.4		0.68 0.41
More than $50,000	32,677	50.7		0.04
Race			<0.001*	
White	63,200	73.4		Ref
Black	8,637	16.0		0.002*
Asian	2,726	4.2		0.15
Other race	5,059	4.7		0.002*
Multiracial	2,329	1.7		0.02*
**Biologic Factors**				
Age			<0.001*	
18 to 44	23,560	44.2		Ref
45 to 54	11,978	14.8		<0.001*
55 to 64	16,233	17.1		<0.001*
65 or older	30,488	23.9		<0.001*
Body-Mass Index (BMI)			<0.001*	
Normal Weight	26,178	35.7		Ref
Underweight	1,584	2.3		0.91
Overweight	22,233	29.5		0.27
Obese	22,573	32.5		<0.001*
**Behavioral Factors**				
Smoking Status			<0.001*	
Never Smoked	50,746	65.5		Ref
Current Smoker	9,817	13.2		0.69
Former Smoker	19,299	21.3		<0.001*
Alcohol Consumption			0.04*	
Not a Heavy Drinker	73,498	93.7		Ref
Heavy Drinker	4,780	6.3		0.04*

*p-value < 0.05 indicating significant results

### Statistical analysis

This survey data required that we account for the sampling probability. Accordingly, we carried out weighted analyses using sampling and cluster weights that adjust for the complex sampling design of the BRFSS. Focusing our analysis on female participants only does not necessitate any recalculation of the weights as the aim of the weighted approach is to guarantee sufficient representation for small sub-samples or regions within the population. Hence keeping the sampling and cluster weights as calculated by CDC should continue to insure sufficient representation of women from different regions irrespective of the region’s size.

Summary statistics (counts and weighted percentages) were first generated for all dependent and independent variables. Frequency distribution analysis was conducted to present the characteristics of the study population stratified as women with and without endometrial cancer, and women with and without diabetes. Crude overall unadjusted associations between the social, biologic and behavioral determinants of health, and endometrial cancer were reported using weighted Chi-square tests. Sub-analysis was also conducted on these determinants of health and diabetes to assess the crude associations between these variables and diabetes. The results were expressed in terms of weighted percentages and p-values. Weighted simple logistic regressions were also carried out to assess the crude association between each level of the predictors and endometrial cancer (the main outcome of the study). Similarly, additional sub-analysis on the determinants of health and diabetes were performed using weighted simple logistic regression to determine the association between each level of the variable and the odds of diabetes relative to a reference category.

Finally, weighted multiple logistic regression was performed on endometrial cancer and the variables that were eligible to be included in the multivariable model. Eligible variables were the ones that had p-values in the weighted simple logistic regression of 0.2 and below. The cutoff level for the eligibility for multiple logistic regression was raised to 0.2 and below since the significance level of 0.05 was shown to fail in an effective selection of covariates that are associated with the outcome [39]. Measures of associations were reported in terms of Cramer’s V, the unadjusted and adjusted Odds Ratios (OR) and the corresponding 95% confidence intervals (CI) of the OR. Data analysis was carried out using the statistical software STATA 18.

### Ethical considerations

We have conducted a quantitative study that undertook secondary analysis on a surveillance data that is publicly available at the CDC website. This study was exempted from IRB submission and approval at the American University of Beirut since it is based on a fully de-identified public access data (BRFSS). Further details on the ethical considerations of this study are included in the Declarations section of this manuscript. All the results reported in this study can be replicated using the weighted analyses described in the Statistical Analysis and the BRFSS dataset with inclusion criteria denoted in the Study Population and Sampling sub-sections respectively.

## Results

### Descriptive statistics

Table 1 displays the characteristics of the 84,118 participants included in the study from the BRFSS data. Of the participants included, 408 women had endometrial cancer, 10,930 had diabetes, and 106 had both endometrial cancer and diabetes. The majority of women owned their home (69.3%, *n* = 58,970), reported that they were married or coupled (52.9%, *n* = 43,625), had healthcare coverage (90.2%, *n* = 77,946), were of the White race (73.4%, *n* = 63,200), lived in urban counties (92.9%, *n* = 73,153) and were employed or self-employed (50.2%, *n* = 37,635). Most of the participants either attended (32.6%, *n* = 24,160), or graduated college/technical school (30.4%, *n* = 33,129), had an income of $50,000 or more (50.7%, *n* = 32,677), and were aged between 18 and 44 years (44.2%, *n* = 23,560). The BMI was almost balanced between the different categories of weight with 35.7% of women reporting having normal weight (*n* = 26,178), 29.5% overweight (*n* = 22,233), 32.5% obese (*n* = 22,573), with only 2.3% underweight (*n* = 1,584). Most of the participants reported that they never smoked (65.5%, *n* = 50,746) and were not heavy drinkers (93.7%, *n* = 73,498).

### Frequency distribution of each level of the different variables among women with and without diabetes

Table 2 displays the frequency distribution of the levels of the different SDOH, biologic, and behavioral determinants among women with and without diabetes mellitus. The weighted chi-squared test results revealed a significant association between diabetes, the main predictor in the study, and the different variables (*p* < 0.001). The majority of women with diabetes owned their home (72.4%), were married or coupled (46.4%), had healthcare coverage (93.2%), were retired (39.1%), lived in urban counties (90.4%), attended college or technical school (31.7%), had an income higher than $50,000 (31.2%), and were of the White race (66.4%). Moreover, most of the women with diabetes were aged 65 or more (46.5%), obese (57.0%), never smoked (58.2%), and not heavy drinkers (97.5%).

**Table 2:** Frequency distribution of each level of the different variables among participants with and without diabetes, and crude associations with diabetes

**Variables**	**Among Women with Diabetes** **N (Weighted %)**^a^	**Among Women without Diabetes** **N (Weighted %)**^b^	**Weighted Chi-square *p*-value**	**Weighted Unadjusted Logistic Regression *P*-value**
**Social Determinants of Health**				
Home Ownership			<0.001*	
Own	7,530 (72.4)	51,440 (68.9)		Ref
Rent	2,767 (23.2)	17,289 (24.4)		0.016*
Other Arrangement	541 (4.4)	3,83 (6.7)		<0.001*
Marital Status			<0.001*	
Married or Coupled	4,662 (46.4)	38,963 (53.7)		Ref
Divorced or Separated	2,218 (20.1)	11,237 (13.3)		<0.001*
Widowed	2,662 (20.3)	9,991 (8.6)		<0.001*
Never Married	1,312 (13.3)	12,291 (24.4)		<0.001*
Health Care Coverage			<0.001*	
No	534 (6.8)	5,307 (10.2)		Ref
Yes	10,355 (93.2)	67,591 (89.8)		<0.001*
Employment Status			<0.001*	
Employed or Self-Employed	2,785 (27.8)	34,850 (53.0)		Ref
Out of Work/Unable to Work	2,248 (24.3)	8,598 (13.6)		<0.001*
Homemaker/Student	793 (8.8)	7,565 (14.7)		0.052
Retired	4,931 (39.1)	20,902 (18.7)		<0.001*
Urban/Rural County			<0.001*	
Urban	9,168 (90.4)	63,985 (93.2)		Ref
Rural	1,572 (9.6)	8,337 (6.8)		<0.001*
Education Level			<0.001*	
Graduated high school	3,423 (31.5)	17,955 (25.9)		Ref
Did not graduate high school	1,262 (19.2)	3,869 (9.4)		<0.001*
Attended college or technical school	3,315 (31.7)	20,845 (32.7)		<0.001*
Graduated college or technical school	2,889 (17.6)	30,240 (32.0)		<0.001*
Income Level			<0.001*	
Less than $15,000	1,407 (17.8)	4,635 (8.5)		Ref
$15,000 to less than $25,000	2,093 (24.8)	8,817 (15.5)		<0.001*
$25,000 to less than $35,000	1,073 (12.0)	5,772 (9.6)		<0.001*
$35,000 to less than $50,000	1,218 (14.2)	7,923 (13.3)		<0.001*
More than $50,000	2,611 (31.2)	30,066 (53.1)		<0.001*
Race			<0.001*	
White	7,209 (66.4)	55,991 (74.3)		Ref
Black	1,819 (23.6)	6,818 (15.1)		<0.001*
Asian	308 (2.7)	2,418 (4.3)		0.014
Other race	943 (5.1)	4,116 (4.6)		0.002
Multiracial	376 (2.2)	1,953 (1.7)		0.004
**Biologic Factors**				
Age			<0.001*	
18 to 44	857 (13.2)	22,703 (48.1)		Ref
45 to 54	1,311 (14.6)	10,667 (14.8)		<0.001*
55 to 64	2,580 (25.7)	13,653 (16.0)		<0.001*
65 or older	5,990 (46.5)	24,498 (21.1)		<0.001*
Body-Mass Index (BMI)			<0.001*	
Normal Weight	1,518 (14.8)	24,660 (38.4)		Ref
Underweight	81 (0.7)	1,503 (2.4)		0.135
Overweight	2,667 (27.5)	19,566 (29.8)		<0.001*
Obese	5,197 (57.0)	17,376 (29.4)		<0.001*
**Behavioral Factors**				
Smoking Status			<0.001*	
Never Smoked	6,082 (58.2)	44,664 (66.5)		Ref
Current Smoker	1,396 (14.6)	8,421 (13.0)		<0.001*
Former Smoker	2,941 (27.2)	16,358 (20.5)		<0.001*
Alcohol Consumption			<0.001*	
Not a Heavy Drinker	10,085 (97.5)	63,413 (93.2)		Ref
Heavy Drinker	221 (2.5)	4,559 (6.8)		<0.001*

^a^Frequency distribution (counts and weighted percentages) of each variable in individuals with diabetes

^b^Frequency distribution (counts and weighted percentages) of each variable in individuals with no diabetes

*p-value < 0.05 indicating significant results

### Diabetes, SDOH, biologic, behavioral factors and endometrial cancer

Table 1, Supplementary Table S1 and Table 3 present the respective unadjusted and adjusted measures of associations between diabetes, SDOH, biologic and behavioral factors, and endometrial cancer expressed in terms of Cramer’s V, unadjusted and adjusted ORs. Weighted Cramer’s V showed mild effect size and magnitude of association between most of the variables and endometrial cancer (Supplementary Table S1). In addition, the unadjusted analysis revealed significant crude associations between majority of the variables and endometrial cancer, except for healthcare coverage, urban/rural areas of residency, education and income levels (Table 1, and Supplementary Table S1). With respect to the adjusted analysis, our results showed that women with diabetes had a 54% increase in the odds of endometrial cancer (approximately double the odds), compared to women without diabetes (OR 1.54; 95%CI: 1.01–2.34).

**Table 3:** Adjusted associations between diabetes, SDOH, biologic, behavioral factors and endometrial cancer

**Endometrial Cancer**	**Weighted Adjusted OR (95% CI)**^┼^	***p*-value**^**┼**^
Diabetes		
No	Ref	
Yes	1.54 (1.01-2.34)*	0.04*
**Social Determinants of Health**		
Home Ownership		
Own	Ref	
Rent	0.50 (0.28-0.88)*	0.02*
Other Arrangement	0.05 (0.02-0.16)*	<0.001*
Marital Status		
Married or Coupled	Ref	
Divorced or Separated	0.55 (0.30-0.99)*	0.05*
Widowed	0.94 (0.58-1.54)	0.82
Never Married	0.97 (0.48-1.96)	0.93
Health Care Coverage		
No	Ref	
Yes	0.61 (0.22-1.70)	0.34
Employment Status		
Employed or Self-Employed	Ref	
Out of Work/Unable to Work	0.99 (0.51-1.92)	0.98
Homemaker/Student	1.23 (0.57-2.64)	0.60
Retired	1.39 (0.76-2.56)	0.28
Urban/Rural County		
Urban	Ref	
Rural	0.92 (0.49-1.72)	0.80
Education Level		
Graduated high school	Ref	
Did not graduate high school	0.71 (0.28-1.81)	0.48
Attended college or technical school	1.83 (1.12-3.00)*	0.02*
Graduated college or technical school	1.53 (0.90-2.60)	0.12
Income Level		
Less than $15,000	Ref	
$15,000 to less than $25,000	0.48 (0.22-1.04)	0.06
$25,000 to less than $35,000	0.47 (0.22-1.02)	0.06
$35,000 to less than $50,000	0.35 (0.16-0.78)*	0.01*
More than $50,000	0.29 (0.14-0.62)*	0.001*
Race		
White	Ref	
Black	0.24 (0.07-0.84)*	0.02*
Asian	1.02 (0.16-6.34)	0.98
Other race	0.37 (0.15-0.88)*	0.02*
Multiracial	0.50 (0.21-1.20)	0.12
**Biologic Factors **		
Age		
18 to 44	Ref	
45 to 54	2.75 (1.01-7.71)*	0.05*
55 to 64	4.20 (1.61-10.92)*	0.003*
65 or older	7.21 (2.76-18.82)*	<0.001*
Body-Mass Index (BMI)		
Normal Weight	Ref	
Underweight	0.44 (0.07-2.57)	0.36
Overweight	1.15 (0.67-1.95)	0.61
Obese	3.10 (1.96-4.90)*	<0.001*
**Behavioral Factors**		
Smoking Status		
Never Smoked	Ref	
Current Smoker	1.30 (0.67-2.52)	0.44
Former Smoker	1.34 (0.86-2.10)	0.20
Alcohol Consumption		
Not a Heavy Drinker	Ref	
Heavy Drinker	0.68 (0.31-1.51)	0.35

^┼^Weighted multiple logistic regression showing the adjusted associations between each level of the variables in comparison with the reference category and endometrial cancer

*p-value ≤ 0.05 indicating significant results

Our adjusted analysis (Table 3) also showed that indices of SDOH and biologic factors had significant associations with endometrial cancer. However, none of the behavioral factors presented a significant association with this type of cancer.

The indices of SDOH that were associated with increased odds of endometrial cancer included the level of education of attending college or technical schools with an 83% associated increase in the respective odds of endometrial cancer compared to the level of education of graduated high school (OR 1.83; 95%CI: 1.12-3.00). Moreover, the biologic factors that were also associated with an increase in the odds of endometrial cancer included older age and obesity. In this regard, women whose ages were between 45 and 54 (OR 2.75; 95%CI: 1.01–7.71), 55 and 64 (OR 4.20; 95%CI: 1.61–10.92), and 65 or older (OR 7.21; 95%CI: 2.76–18.82) were shown to have about 3- to 7-fold increase in the estimated risk of endometrial cancer compared to the younger reference age group of 18 to 44. In addition, women who were considered obese were 3 times more likely to have endometrial cancer compared to women of normal weight (OR 3.10; 95%CI: 1.96–4.90).

On the other hand, the SDOH that were associated with a decrease in the odds of endometrial cancer compared to their respective reference categories (indicated in Table 3) included women who reported renting a home or had other arrangements for homeownership, were divorced or separated, had higher ranges of income, were of Black or other races. In this regard, renting a home (OR 0.50; 95%CI: 0.28–0.88) or having other arrangements (OR 0.05; 95%CI: 0.02–0.16) for statuses of home ownership were associated with 50 and 95% lower odds of endometrial cancer compared to owning a home. Moreover, being divorced or separated as marital status was shown to be associated with a 45% decrease in the odds of endometrial cancer (OR 0.55; 95%CI: 0.30–0.99) compared to being married or coupled. Along the same lines, a higher annual income of $35,000 to $50,000 (OR 0.35; 95%CI: 0.16–0.78), and $50,000 or more (OR 0.29; 95%CI: 0.14–0.62) were income categories that were associated with respective 65 and 71% decrease in the odds of endometrial cancer compared to the lower income category of less than $15,000. As for race, Black women (OR 0.24; 95%CI: 0.07–0.84) and women of other races (OR 0.37; 95%CI: 0.15–0.88) showed respective decreases of 66 and 63% in the odds of endometrial cancer compared to White women.

Our main multivariable analysis was followed by a number of confirmatory additional analyses that took into consideration several conditions.

First, given the cross-sectional nature of our study design, we anticipated that some women might have had endometrial cancer before diabetes. Accordingly, we performed additional multivariable analysis in which we excluded women (32 women in total) who were diagnosed with endometrial cancer before their diagnosis with diabetes. Our results (not shown for all the variables) were not substantially affected by this left censoring, and diabetes was still significantly associated with endometrial cancer (OR 1.81; 95%CI: 1.11–2.94; *p* = 0.017).

Then we carried out new analyses in which we incorporated the age of diagnosis with diabetes in one multivariable model, and the duration of diabetes in another multivariable model, with study population being exclusive to women with diabetes (results not shown for all the variables). Our results showed that age of diagnosis with diabetes (OR 0.97; 95%CI: 0.95-1.00; *P* = 0.104), and duration of diabetes (OR 1.02; 95%CI: 0.99–1.05; *p* = 0.108) did not have significant associations with endometrial cancer.

Our original analysis assumed that the control group is comprised of women who specifically did not have endometrial cancer. To confirm these denoted associations, we conducted further analysis in which we considered our control group as women who did not have any type of cancer (results not reported for all the variables). The new results showed that diabetes continued to be a significant predictor of endometrial cancer with OR = 1.54, 95%CI: 1.01–2.35, *P* = 0.043.

Lastly, we carried out additional sub-analysis that focused on women who reported having more than one type of cancer and age of diagnosis was recorded for the first type of cancer (total of 2854 women). In this analysis we aimed to determine if age of diagnosis with other types of cancer was associated with the odds of endometrial cancer, along with our main predictors which included diabetes and determinants of health. Results of this analysis are presented in Table 4 and showed that age of diagnosis with other types of cancer was not significantly associated with endometrial cancer *P* = 0.18, but a significant association was still present between diabetes and endometrial cancer in this subpopulation of women (OR = 2.28, 95%CI: 1.02–5.12, *P* = 0.04).

**Table 4:** Adjusted associations between diabetes, SDOH, biologic, behavioral factors, and endometrial cancer for participants with more than type of cancer including the age of diagnosis with the first type of cancer

**Endometrial Cancer**	**Weighted Adjusted OR (95% CI)**^┼^	***p*-value**^**┼**^
Age of Diagnosis with Cancer	0.97 (0.94-1.01)	0.18
Diabetes		
No	Ref	
Yes	2.28 (1.02-5.12)*	0.04*
**Social Determinants of Health**		
Home Ownership		
Own	Ref	
Rent	1.01 (0.36-2.78)	0.98
Other Arrangement	0.14 (0.01-2.03)	0.15
Marital Status		
Married or Coupled	Ref	
Divorced or Separated	0.09 (0.02-0.44)*	<0.01*
Widowed	0.58 (0.18-1.84)	0.36
Never Married	0.60 (0.10-3.59)	0.58
Health Care Coverage		
No	Ref	
Yes	4.99 (0.41-60.23)	0.21
Employment Status		
Employed or Self-Employed	Ref	
Out of Work/Unable to Work	0.27 (0.06-1.16)	0.08
Homemaker/Student	2.38 (0.59-9.60)	0.22
Retired	1.33 (0.39-4.44)	0.64
Urban/Rural County		
Urban	Ref	
Rural	1.26 (0.31-5.10)	0.74
Education Level		
Graduated high school	Ref	
Did not graduate high school	0.60 (0.08-4.57)	0.62
Attended college or technical school	1.87 (0.56-6.23)	0.30
Graduated college or technical school	1.18 (0.32-4.32)	0.79
Income Level		
Less than $15,000	Ref	
$15,000 to less than $25,000	0.17 (0.03-0.88)	0.03*
$25,000 to less than $35,000	0.27 (0.04-1.66)	0.16
$35,000 to less than $50,000	0.10 (0.02-0.56)*	0.01*
More than $50,000	0.09 (0.01-0.55)*	0.01*
Race		
White	Ref	
Black	0.37 (0.05-2.94)	0.35
Asian	Omitted no cell counts	
Other race	0.30 (0.02-4.38)	0.38
Multiracial	0.65 (0.09-4.69)	0.67
**Biologic Factors **		
Age		
18 to 44	Ref	
45 to 54	0.97 (0.09-10.21)	0.98
55 to 64	1.21 (0.19-7.47)	0.83
65 or older	1.06 (0.13-8.14)	0.95
Body-Mass Index (BMI)		
Normal Weight	Ref	
Underweight	0.16 (0.01-1.71)	0.13
Overweight	0.84 (0.26-2.66)	0.77
Obese	1.85 (0.59-5.73)	0.28
**Behavioral Factors**		
Smoking Status		
Never Smoked	Ref	
Current Smoker	0.33 (0.07-1.59)	0.17
Former Smoker	0.63 (0.26-1.52)	0.31
Alcohol Consumption		
Not a Heavy Drinker	Ref	
Heavy Drinker	0.98 (0.17-5.45)	0.98

^┼^Weighted multiple logistic regression showing the adjusted associations between each level of the variables in comparison with the reference category and endometrial cancer

*p-value ≤ 0.05 indicating significant results

In addition to identifying diabetes as a significant predictor of endometrial cancer in all of the aforementioned analyses, our multivariable models also revealed strong relationships between this type of cancer and several determinants of health. These indices included, but were not limited to, homeownership, marital status, education, income, age and BMI; thus, confirming the link between these determinants of health and endometrial cancer.

## Discussion

In the present study, we examined the cumulative effect of a cluster of covariates that included diabetes, SDOH, behavioral, and biologic factors on endometrial cancer among women in the US. Our findings indicated that diabetes, biologic factors (age and obesity) and social and racial determinants are associated with the risk of occurrence and development of endometrial cancer.

Our analysis revealed that women with diabetes had almost double the estimated risk of endometrial cancer compared to women without diabetes. This detected association could be attributed to hyperinsulinemia in type 2 diabetes that contributes to elevated estrogen levels and consequently the development of endometrial cancer [5, 9]. Our result is consistent with previous findings which reported an increased risk of endometrial cancer among diabetic women with risk ratios ranging between 1.7 and 2.1 [7–15].

Our analysis also revealed significant associations between biologic factors (age and obesity), as well as indices of social determinants of health (SDOH), with this type of cancer. However, it did not detect significant associations between behavioral factors (smoking and heavy alcohol consumption) and endometrial cancer. The undetected association is not surprising since the literature had inconsistent findings concerning the effect of smoking and heavy alcohol consumption on the risk of endometrial cancer [23, 26–28, 40]. Our results indicated that older age and obesity were more prevalent among women with diabetes and were significantly associated with increased odds of endometrial cancer. Among the SDOH, our data indicated that the level of education of attending college or technical school was also associated with increased odds of endometrial cancer and a higher prevalence of this level of education among women with diabetes. This observation can be driven by the fact that people with lower levels of education tend to miss more on preventative checkup visits and available screening facilities [41], which may contribute to under-reporting of cases among individuals with lower levels of education compared to those who are more educated.

Conversely, individuals who rented their homes, had other living arrangements, were divorced, had incomes over $35,000, and were of Black or other race had lower odds of endometrial cancer and were less prevalent among women with diabetes compared to their respective reference categories. However, the result pertinent to Black women need to be carefully interpreted in view of the uterine cancer high mortality rate in this group of women [42]. In particular, Black women were shown to be more prone of diagnosis with more aggressive forms of endometrial cancer compared to other races, resulting in an increased disease-attributed mortality rate among this group of women [42]. This may lead to an under-representation of Black women in the BRFSS study that are missed either due to the severity of their endometrial cancer related illness, or death.

Accordingly, our results highlighted the relationship between diabetes, biologic and social determinants of health, with endometrial cancer, and indicated that the discrepancies in the diagnosis of diabetes and the incidence of endometrial cancer may be related to racial and socioeconomic differences.

One potential explanation for the effect of age and obesity on endometrial cancer could be related to menopause and high levels of body fat. Menopause, a physiological change that typically occurs among women of older age, is known to be associated with the growth of the tissues in the lining of the uterus into its muscular wall, increasing the risk of endometrial cancer [3]. In addition, hormone replacement therapies (unopposed estrogen, combined estrogen and progesterone, or tibolone) used by women having menopausal symptoms [20] were shown to double the risk of endometrial cancer [22]. Fatty tissues that result from obesity promote higher levels of estrogen which contribute to the development of endometrial cancer [5]. In addition, high levels of fat in the abdomen observed during menopausal years, combined with older age, can also increase the risk of endometrial cancer [43].

Furthermore, the observed social and racial disparities in endometrial cancer may be linked to differences in healthcare access, screening facilities, and quality of care [5] which ultimately reflect on the detection and reporting of new cancer cases in the different socioeconomic and racial groups. For instance, compared to White women, Black women were found to have less healthcare coverage and limited access to preventive medical care such as screening and genetic testing [5]. Married women were reported to have earlier diagnoses with better prognoses for this type of cancer and greater compliance with regular medical checkups in general, compared to non-married women [32]. This can due to the mental, social, and financial support provided by their spouses [32, 44–46]. Awareness of the symptoms and risk factors for this disease, and its early diagnosis, are usually coupled with higher levels of education and financial stability that enable women to lead a healthier lifestyle and have better access to preventive measures and quality of care [30, 47–49].

## Limitations

Despite the novelty of the data presented in our study, there are some limitations that need to be highlighted. First, given the cross-sectional nature of the BRFSS, all the detected relationships should be interpreted as associations and not as causal-effect inferences. Moreover, the odds ratios reported in this study can be viewed as prevalence odds ratio since endometrial cancer might have occurred before diabetes. However, this was not a major concern in our study since only very few women were diagnosed with endometrial cancer before their diagnosis with diabetes. This issue was addressed in the additional analysis that we performed in which we excluded this group of women, and the results aligned with all the associations that were detected in our original analysis. The study population of the BRFSS may be more representative of women who survived their endometrial cancer, and less reflective of women with more aggressive forms of this disease. The latter group may have been under-represented either because women were too ill to respond or have passed away due to endometrial cancer related mortality. Thus, this surveillance system might have missed on women who were diagnosed further in the past and subsequently died, and may have captured more, women with less severe past diagnosis of endometrial cancer. Missing out on potential severe cases may bias our results and study population towards more survivorship than the overall general population of endometrial cancer.

In response to the survey, 22 states agreed to administer the questions on cancer survivorship, while the remaining states declined to participate in this part of the questionnaire. This for sure imposes some limitation on the generalizability of the results. In addition, our data had only 106 women who had both diabetes and endometrial cancer, which can also pose some limitation on the inferences. However, the fact that our results showed a consistent association between diabetes and endometrial cancer, that continued to prevail despite of the large denominator of 84,118 female participants, comes in support of the presence of a strong relationship between these two comorbid diseases. Lastly, it is important to note that the BRFSS data might be subject to recall and social desirability bias due to the self-reported information. Nonetheless, validity of this data was underscored in the context of studies where self-reporting entailed easy-to-understood questions, secured anonymity, and full absence of reprisal [50]. These settings mirror the context of the data collection process that was undertaken in the BRFSS study.

## Conclusion

In conclusion, our study is the first to adopt a comprehensive approach to the assessment of the effect of diabetes, SDOH, biologic and behavioral factors on endometrial cancer using nationally representative data. In this study we provided further evidence that underlie the growing burden of diabetes with increased risk for endometrial cancer progression. Our data also point to the social and racial disparities associated with poor prognosis of women with endometrial cancer. In light of our data and with the increasing incidence rates, endometrial cancer is set to become a significant public health problem [7] which necessitates corrective measures at the level of modifiable risk factors such as the ones we addressed in our study. The observed social disparities in the health outcomes of endometrial cancer can be reconciled by adopting policies and social structures that endorse standardized early detection management programs and preventative strategies covered by the healthcare system and advocate for equitable access to healthcare services. Active implementation of such recommendations is key for addressing social and racial inequities in health and reducing the burden of this cancer among women. Future studies could further explore the mechanisms through which diabetes, and social determinants of health modulate biologic markers on risk to develop endometrial cancer.

### Supplementary Information

**Supplementary Material 1.**

## Data Availability

Dataset is publicly available on the CDC website, and can also be provided by the corresponding authors upon request.

## References

[1] NCI. Endometrial cancer. 2023. https://www.cancer.gov/publications/dictionaries/cancer-terms/def/endometrial-cancer. Accessed.

[2] Crosbie EJ, Kitson SJ, McAlpine JN, Mukhopadhyay A, Powell ME, Singh N (2022). Endometrial cancer. Lancet.

[3] Wild CP, Weiderpass E (2020). World cancer report: cancer research for cancer prevention.

[4] Xia C, Dong X, Li H (2022). Cancer statistics in China and United States, 2022: profiles, trends, and determinants. Chin Med J (Engl).

[5] Makker V, MacKay H, Ray-Coquard I (2021). Endometrial cancer (primer). Nat Rev Dis Primers.

[6] WHO. Diabetes. 2023. https://www.who.int/news-room/fact-sheets/detail/diabetes. Accessed.

[7] Lu KH, Broaddus RR (2020). Endometrial cancer. N Engl J Med.

[8] Wang Y, Zeng X, Tan J, Xu Y, Yi C (2022). Diabetes mellitus and endometrial carcinoma: risk factors and etiological links. Medicine.

[9] Njoku K, Agnew HJ, Crosbie EJ (2022). Impact of type 2 diabetes mellitus on endometrial cancer survival: a prospective database analysis. Front Oncol.

[10] Gallagher EJ, LeRoith D (2015). Obesity and diabetes: the increased risk of cancer and cancer-related mortality. Physiol Rev.

[11] Suh S, Kim KW (2019). Diabetes and cancer: cancer should be screened in routine diabetes assessment. Diabetes Metab J.

[12] Tsilidis KK, Kasimis JC, Lopez DS, Ntzani EE, Ioannidis JP (2015). Type 2 diabetes and cancer: umbrella review of meta-analyses of observational studies. BMJ.

[13] Saed L, Varse F, Baradaran HR (2019). The effect of diabetes on the risk of endometrial Cancer: an updated a systematic review and meta-analysis. BMC Cancer.

[14] McVicker L, Cardwell CR, Edge L, McCluggage WG, Quinn D, Wylie J, McMenamin UC (2022). Survival outcomes in endometrial cancer patients according to diabetes: a systematic review and meta-analysis. BMC Cancer.

[15] Liao C, Zhang D, Mungo C, Andrew Tompkins D, Zeidan AM (2014). Is diabetes mellitus associated with increased incidence and disease-specific mortality in endometrial cancer? A systematic review and meta-analysis of cohort studies. Gynecol Oncol.

[16] Yeh HC, Golozar A, Brancati FL. Cancer and Diabetes. In: Cowie CC, Casagrande SS, Menke A, Cissell MA, Eberhardt MS, Meigs JB, Gregg EW, Knowler WC, Barrett-Connor E, Becker DJ, Brancati FL, Boyko EJ, Herman WH, Howard BV, Narayan KMV, Rewers M, Fradkin JE, editors. Diabetes in America. 3rd ed. Bethesda: National Institute of Diabetes and Digestive and Kidney Diseases (US); 2018. CHAPTER 29. PMID: 33651571. 33651571

[17] Chobot A, Górowska-Kowolik K, Sokołowska M, Jarosz-Chobot P (2018). Obesity and diabetes-not only a simple link between two epidemics. Diabetes Metab Res Rev.

[18] Shaw E, Farris M, McNeil J (2016). Obesity and endometrial cancer. Recent Results Cancer Res.

[19] NCI. Cancer stat facts: uterine cancer. 2023. https://seer.cancer.gov/statfacts/html/corp.html. Accessed.

[20] North American Menopause Society. The North American menopause society statement on continuing use of systemic hormone therapy after Age 65. Menopause. 2015;22(7):693. 10.1097/GME.0000000000000492. 10.1097/GME.0000000000000492 26035150

[21] NIH. What is menopause? 2021. https://www.nia.nih.gov/health/what-menopause#:~:text=The%20menopausal%20transition%20most%20often,begins%2C%20and%20race%20and%20ethnicity. Accessed.

[22] Sjögren LL, Mørch LS, Løkkegaard E (2016). Hormone replacement therapy and the risk of endometrial cancer: a systematic review. Maturitas.

[23] Zhou B, Yang L, Sun Q (2008). Cigarette smoking and the risk of endometrial cancer: a meta-analysis. Am J Med.

[24] Felix AS, Yang HP, Gierach GL, Park Y, Brinton LA (2014). Cigarette smoking and endometrial carcinoma risk: the role of effect modification and tumor heterogeneity. Cancer Causes Control: CCC.

[25] Dimou N, Omiyale W, Biessy C (2022). Cigarette smoking and endometrial cancer risk: observational and mendelian randomization analyses. Cancer Epidemiol Biomarkers Prev.

[26] Zhou Q, Guo P, Li H, Chen XD (2017). Does alcohol consumption modify the risk of endometrial cancer? A dose-response meta-analysis of prospective studies. Arch Gynecol Obstet.

[27] Sun Q, Xu L, Zhou B, Wang Y, Jing Y, Wang B (2011). Alcohol consumption and the risk of endometrial cancer: a meta-analysis. Asia Pac J Clin Nutr.

[28] Turati F, Gallus S, Tavani A (2010). Alcohol and endometrial cancer risk: a case-control study and a meta-analysis. Cancer Causes Control: CCC.

[29] Wang Q, Wang R, Chen C (2022). Educational attainment and endometrial cancer: a mendelian randomization study. Front Genet.

[30] Svanvik T, Marcickiewicz J, Sundfeldt K (2019). Sociodemographic disparities in stage-specific incidences of endometrial cancer: a registry-based study in West Sweden, 1995–2016. Acta Oncol.

[31] Whetstone S, Burke W, Sheth SS (2022). Health disparities in uterine cancer: report from the uterine cancer evidence review conference. Obstet Gynecol.

[32] Dong J, Dai Q, Zhang F (2019). The effect of marital status on endometrial cancer-related diagnosis and prognosis: a surveillance epidemiology and End results database analysis. Future Oncol (London England).

[33] Mukerji B, Baptiste C, Chen L (2018). Racial disparities in young women with endometrial cancer. Gynecol Oncol.

[34] Felix AS, Weissfeld JL, Stone RA (2010). Factors associated with type I and type II endometrial cancer. Cancer Causes Control: CCC.

[35] CDC. Cancer statistics data visualizations tool, based on 2021 submission data (1999–2019). 2022. https://gis.cdc.gov/Cancer/USCS/#/Demographics/. Accessed.

[36] CDC. 2020 BRFSS survey data and documentation. 2021. https://www.cdc.gov/brfss/annual_data/annual_2020.html. Accessed 11 Apr 2022.

[37] CDC. Behavioral risk factor surveillance system. 2021. https://www.cdc.gov/brfss/index.html. Accessed.

[38] CDC. Defining adult overweight & obesity. 2021. https://www.cdc.gov/obesity/basics/adult-defining.html?CDC_AA_refVal=https%3A%2F%2Fwww.cdc.gov%2Fobesity%2Fadult%2Fdefining.html. Accessed.

[39] Bursac Z, Gauss CH, Williams DK, Hosmer DW (2008). Purposeful selection of variables in logistic regression. Source Code Biol Med.

[40] Raglan O, Kalliala I, Markozannes G (2019). Risk factors for endometrial cancer: an umbrella review of the literature. Int J Cancer.

[41] Castro S, Sosa E, Lozano V, Akhtar A, Love K, Duffels J, Raz JD, Kim YJ, Sun V, Erhunmwunsee L (2021). The impact of income and education on lung cancer screening utilization, eligibility, and outcomes: a narrative review of socioeconomic disparities in lung cancer screening. J Thorac Dis.

[42] Clarke MA, Devesa SS, Hammer A, et al. Racial and ethnic differences in hysterectomy-corrected uterine corpus cancer mortality by stage and histologic subtype. JAMA Oncol. 2022;5. 10.1001/jamaoncol.2022.0009. 10.1001/jamaoncol.2022.0009 PMC9073658 35511145

[43] Dunneram Y, Greenwood DC, Cade JE (2019). Diet, menopause and the risk of ovarian, endometrial and breast cancer. The Proceedings of the Nutrition Society.

[44] Buja A, Lago L, Lago S, Vinelli A, Zanardo C, Baldo V. Marital status and stage of cancer at diagnosis: A systematic review. Eur J Cancer Care (Engl). 2018;27(1). 10.1111/ecc.12755. 10.1111/ecc.12755 28850741

[45] Baine M, Sahak F, Lin C, Chakraborty S, Lyden E, Batra SK (2011). Marital status and survival in pancreatic cancer patients: a SEER based analysis. PLoS ONE.

[46] Hanske J, Meyer CP, Sammon JD (2016). The influence of marital status on the use of breast, cervical, and colorectal cancer screening. Prev Med.

[47] McDaniel JT, Nuhu K, Ruiz J, Alorbi G (2019). Social determinants of cancer incidence and mortality around the world: an ecological study. Glob Health Promot.

[48] Donkers H, Bekkers R, Massuger L, Galaal K (2019). Systematic review on socioeconomic deprivation and survival in endometrial cancer. Cancer Causes Control.

[49] Kucera CW, Tian C, Tarney CM, Presti C, Jokajtys S, Winkler SS (2023). Factors associated with survival disparities between non-hispanic black and white patients with uterine cancer. JAMA Netw Open.

[50] Brener ND, Billy JOG, Grady WR (2003). Assessment of factors affecting the validity of self-reported health-risk behavior among adolescents: evidence from the scientific literature [pdf 200K]. J Adolesc Health.

