# Supplementary material for: Diabetes and the social, biologic, and behavioral determinants of endometrial cancer in the United States
Source: BMC Cancer. 2024 Apr 29;24:540. doi: 10.1186/s12885-024-12192-y (PMC11057164; doi:10.1186/s12885-024-12192-y)
Supplement: Supplementary file 1 — Supplementary Material 1. [file 12885_2024_12192_MOESM1_ESM.docx]

**Supplementary Table S1 Unadjusted associations between diabetes, SDOH, biologic, behavioral factors, and endometrial cancer (outcome), along with the frequency distribution of the levels of the variables among women with endometrial cancer**

| **Endometrial Cancer** | **N** | **Weighted Unadjusted OR**  **(95% CI)^ɸ^** | **Weighed**  **Cramér's V** | **Weighted Chi-square**  **p-value** | **p-value ^ɸ^** |
| --- | --- | --- | --- | --- | --- |
| Diabetes  Yes  No | 302  106 | Ref  3.15 (2.18-4.55)* | 0 .033 | <0.001* | Ref  <0.001* |
| **Social Determinants of Health** |  |  |  |  |  |
| Home Ownership  Own  Rent  Other Arrangement | 328  67  12 | Ref  0.40 (0.25-0.63)*  0.08 (0.03-0.18)* | 0.024 | <0.001* | Ref  <0.001*  <0.001* |
| Marital Status  Married or Coupled  Divorced or Separated  Widowed  Never Married | 200  67  98  40 | Ref  0.67 (0.43-1.06)  1.81 (1.23-2.68)*  0.27 (0.17-0.43)* | 0.031 | <0.001* | Ref  0.08  0.003*  <0.001* |
| Health Care Coverage  No  Yes | 13  395 | Ref  1.96 (0.83-4.59) | 0.011 | 0.11 | Ref  0.12 |
| Employment Status  Employed or Self-Employed  Out of Work/Unable to Work  Homemaker/Student  Retired | 104  54  26  222 | Ref  2.09 (1.27-3.42)*  1.29 (0.70-2.39)  5.24 (3.57-7.68)* | 0.048 | <0.001* | Ref  0.004*  0.41  <0.001* |
| Urban/Rural County  Urban  Rural | 354  52 | Ref  1.53 (0.88-2.65) | 0.008 | 0.12 | Ref  0.13 |
| Education Level  Graduated high school  Did not graduate high school  Attended college or technical school  Graduated college or technical school | 85  22  137  164 | Ref  1.13 (0.56-2.28)  1.45 (0.97-2.17)  0.90 (0.60-1.33) | 0.012 | 0.11 | Ref  0.73  0.07  0.60 |
| Income Level  Less than $15,000  $15,000 to less than $25,000  $25,000 to less than $35,000  $35,000 to less than $50,000  More than $50,000 | 33  53  42  52  150 | Ref  0.71 (0.35-1.43)  0.87 (0.43-1.73)  0.75 (0.37-1.50)  0.55 (0.31-0.98)* | 0.014 | 0.18 | Ref  0.34  0.68  0.41  0.04 |
| Race  White  Black  Asian  Other race^1^  Multiracial | 355  12  4  16  11 | Ref  0.20 (0.07-0.55)*  0.29 (0.05-1.56)  0.27 (0.12-0.61)*  0.36 (0.15-0.82)* | 0.026 | <0.001* | Ref  0.002*  0.15  0.002*  0.02* |

| ***Supplementary Table S1 Unadjusted associations between diabetes, SDOH, biologic, behavioral factors, and endometrial cancer (outcome), along with the frequency distribution of the levels of the variables among women with endometrial cancer (continued)*** | | | | | |
| --- | --- | --- | --- | --- | --- |
| **Endometrial Cancer** | **N** | **Weighted**  **Unadjusted OR**  **(95% CI) ^ɸ^** | **Weighted**  **Cramér's V** | **Weighted Chi-square**  **p-value** | **p-value ^ɸ^** |
| **Biologic Factors** |  |  |  |  |  |
| Age  18 to 44  45 to 54  55 to 64  65 or older | 16  38  81  270 | Ref  4.94 (2.13-11.48)*  7.49 (3.49-16.07)*  16.14 (7.83-33.30)* | 0.058 | <0.001* | Ref  <0.001*  <0.001*  <0.001* |
| Body-Mass Index (BMI)  Normal Weight  Underweight  Overweight  Obese | 87  5  89  177 | Ref  0.92 (0.22-3.83)  1.30 (0.81-2.10)  2.82 (1.83-4.35)* | 0.029 | <0.001* | Ref  0.91  0.27  <0.001* |
| **Behavioral Factors** |  |  |  |  |  |
| Smoking Status  Never Smoked  Current Smoker  Former Smoker | 244  38  123 | Ref  0.89 (0.52-1.55)  1.90 (1.35-2.68)* | 0.019 | <0.001* | Ref  0.69  <0.001* |
| Alcohol Consumption  Not a Heavy Drinker  Heavy Drinker | 391  14 | Ref  0.47 (0.23-0.97)* | 0.010 | 0.04* | Ref  0.04* |

^ɸ^Weighted simple logistic regression showing the unadjusted associations between each level of the variable in comparison with the reference category and endometrial cancer;

*p-value ≤ 0.05 indicating significant results;
